# Development of a scale for measuring the perception of artificial intelligence among mental health consumers

**DOI:** 10.1371/journal.pone.0354305

**Published:** 2026-07-30

**Authors:** Reema Al-Daraiseh, Wafa’a Ta’an, Tareq Mukattash, Mohammed M. Al-Hammouri, Rana Abu-Farha, Brett Williams

**Affiliations:** 1 Faculty of Medicine, Jordan University of Science and Technology, Irbid, Jordan; 2 Faculty of Nursing, Jordan University of Science and Technology, Irbid, Jordan; 3 Faculty of Pharmacy, Jordan University of Science and Technology, Irbid, Jordan; 4 Faculty of Pharmacy, Applied Science Private University, Amman, Jordan; 5 Department of Paramedicine, Monash University, Clayton, Victoria, Australia; Yarmouk University, JORDAN

## Abstract

**Background:**

Artificial Intelligence (AI) has emerged as a transformative force revolutionizing various sectors, including healthcare, particularly the mental health field. However, the acceptance and integration of AI technologies in different healthcare systems can be influenced by various factors, including cultural, social, and individual aspects. Nevertheless, there is a need for a valid and reliable tool for assessing AI’s perception among healthcare consumers.

**Aim:**

To develop and validate a tool for the perception of AI among healthcare consumers and apply the tool to assess AI’s perception among mental health consumers in the Jordanian healthcare system.

**Method:**

A cross‐sectional descriptive correlational design was utilized in the study. Data was collected from a convenience sample of 431 mental health consumers visiting mental health clinics of the International Medical Corps and university hospitals in Jordan. Structured interviews were conducted using an AI Perception (AIP) questionnaire developed by the authors. The questionnaire’s content validity was assessed by an expert panel. Using Principal Component Analysis (PCA), the construct validity of the tool was evaluated, and its internal consistency was examined using Cronbach’s alpha. Descriptive statistics were used to assess the levels of AI perception among participants.

**Results:**

The final AIP tool consisted of 20 items across 4 domains and has demonstrated strong internal consistency across its four domains: AI acceptance and readiness (α = 0.92), AI perceived importance (α = 0.92), AI perceived risk (α = 0.9), and AI perceived challenges (α = 0.85). The construct validity of the four-domain structure of the tool was supported by PCA. Additionally, the mean scores for each domain indicated the average level of agreement with AI perception items among participants. Specifically, the mean score for AI acceptance and readiness was (2.7 ± 0.96). AI perceived importance was (2.18 ± 0.83), AI perceived risk was (2.58± 0.92), and AI perceived challenge was (2.78 ±  0.87).

**Conclusion:**

The findings of this study resulted in developing a valid and reliable 20-item tool to assess AI’s perception among mental health consumers. The tool can be used to assess the predictors of AI’s readiness among mental health consumers. Therefore, aiding policymakers and other stakeholders in understanding the AI adoption barriers from the perspective of end-users. In addition, this study developed the AIP tool that can be validated and used among other populations in future research.

## Introduction

Healthcare is among the top industries that benefit from AI technologies and applications, mostly because the demand for healthcare services is always high and exceeds the resources. Accordingly, there has been a surge in AI applications in the healthcare sector in recent years. The effective use of AI in clinical settings provides an important opportunity for deliberate engagement, especially with the current rapid expansion of the facilities that provide healthcare services, and the utilization of AI tools and applications almost everywhere [1].

The applications of AI in healthcare include clinical decision support systems, which integrate AI with Electronic Health Records (EHRs) to provide recommendations or guidance regarding any step of clinical care. Such an application ensures the provision of safe, effective, and efficient interventions at the point of care. Another example is CURATE.AI, which is an AI-based dosing approach that uses patient-specific data taken over multiple points in time, offering the advantage of adjusting drug doses for one or multiple drugs based on the collected data [2]. This AI dosing model offers great potential for improving patient monitoring regarding responsiveness to treatment.

Health professionals and stakeholders, such as caregivers, managers, and technology companies, are recognizing the growing importance of AI in the future of health care, with each of them having unique interests that drive advancements in the field [3]. Nevertheless, the voices of essential stakeholders, such as service end-users (i.e., patients, caregivers, and families), still seem to be absent from the ongoing discourse about AI in mental health.

There has been tremendous growth in AI applications in the field of mental health in recent years [4], ranging from early detection of mental health disorders [5], developing care plans [6], improved consumer monitoring [7], and multiple other applications. The potential for AI applications in the field of mental health has recently been a major focus, especially in light of the recent increased global demand for mental health services, particularly after the pandemic [8]. Several papers have focused on AI and technology applications in the mental health field because, even though AI and technology applications are being widely adopted in physical health fields as previously discussed, their adoption in the mental health field has been slower [9].

Despite the clear intention for AI technology adoption in health in Jordan, it is important to understand and assess the factors that influence a complete and successful adoption. This topic has been studied thoroughly, and those factors were identified and analyzed over multiple levels. According to The Adoption of AI in the Healthcare Industry Model by [10], five dimensions influence the adoption of AI in the healthcare industry. These dimensions are divided into external antecedents (i.e., technological, regulatory, and macroeconomic readiness) and internal dimensions (i.e., organizational and individual readiness). Unfortunately, previous research highlighted that a substantial portion of patients are still hesitant to integrate AI technologies into their healthcare activities [11].

The questionable readiness for the adoption of new technologies by end users has always been a roadblock to successful implementation. To address this challenge, researchers have worked continuously to theorize and contextualize why end users may accept or reject new technologies. While there has been a consensus that willingness and intention are the main antecedents of the consumers’ actual usage behavior [12], studying potential factors influencing intention and willingness is more complex. However, a major gap in the literature is the lack of published, valid, and reliable instruments to examine the perception of artificial intelligence among mental health consumers.

**Fig 1:**
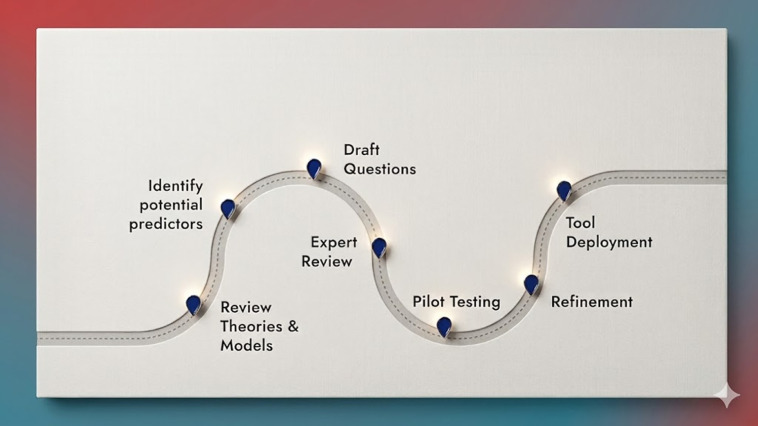
Building the AIP tool.

The aim of this study is to develop and validate a tool to assess the perception of AI among mental health consumers. After tool development, this study will utilize the tool to assess AI’s perceived advantages and challenges from the perspectives of mental health consumers in Jordan. To achieve this, a reliable multi-step protocol was used to build a tool that quantifies AI readiness informed by the framework proposed by [13] (Fig 1). First, a thorough review was conducted on the relevant theories and models to identify a set of potential predictors. The identified predictors were listed, and a group of items were suggested. The final questionnaire draft was reviewed by mental health academics and language experts for validity. Pilot testing was performed with a small sample to assess reliability and internal consistency, and the questionnaire was refined based on the feedback and results. Finally, the final version of the tool, named the AI Perception (AIP) questionnaire, was deployed and tested among mental health consumers.

### Theoretical background

Many theories and models were developed or adapted to assess the predictors of end-users’ perceptions (e.g., readiness and acceptance) towards new technologies, the earliest of which was the Theory of Planned Behavior (TPB). In this theory, Ajzen [14] argues that the attitude towards a behavior, which refers to the individual’s assessment and perception of the behavior, is a main predictor for users’ readiness to adopt this new behavior; in other words, individuals are more likely to perform a certain behavior if they have a positive attitude towards it.

Later, the Technology Acceptance Model (TAM) was developed [15] which builds on the TPB but focuses specifically on the adoption of new technologies. This model theorizes that the intention to use new technology is a function of two main predictors: perceived usefulness and perceived ease of use. In the context of our tool, perceived ease of use corresponds to perceived ability, while perceived benefits align with perceived usefulness, both of which were incorporated as distinct domains to reflect TAM’s framework.

According to the Value-based Adoption Model (VAM), the acceptance and intention of individuals to adopt new technologies are dependent on their perceived value. which in turn, is a function of the balance between perceived benefits and perceived sacrifices or risks. Therefore, the AIP tool includes both perceived benefits and perceived risks to capture this evaluative process. Additionally, the Perceived Risk Theory [16] supports the inclusion of the perceived risk domain by emphasizing that when people face a choice—such as adopting a new technology, making a purchase, or using AI in healthcare—they evaluate the possible risks before shaping their decision.

Literature has also shown that future expectancy or future orientation can influence the readiness and acceptance of new technologies [17]. For example, previous research showed that individuals who are more future-oriented have better chances of using and enjoying new technologies [18], which informed the inclusion of the future directions domain in our tool.

Based on the integration of the above-mentioned theories, the AIP tool was developed to assess multiple interrelated constructs that influence the perception of AI in healthcare. The final domains of the tool were attitude, perceived importance, perceived benefits, perceived ability, perceived risk, and future directions, which together provide a multidimensional framework rooted in established behavioral and technology acceptance theories.

## Methodology

The study’s objectives were to develop and validate a tool to assess AI perception among mental health consumers. A quantitative cross-sectional design was utilized. Since there is no published valid and reliable tool available to assess AI perception among mental health consumers, the authors developed a custom tool guided by the insights from existing literature on the acceptance of new technologies, in addition to experts’ inputs, including mental health professionals and consumer advocates. The questionnaire was developed systematically and in two languages: Arabic and English. Since Arabic is the national language of Jordan, the questionnaire was prepared and circulated in Arabic, ensuring the responses were valid and with minimal misunderstandings.

To establish the validity of the content, a panel of 5 experts in mental health and health informatics reviewed the question set to assess the relevance, comprehensiveness, and clarity of each question using a 4-point scale (i.e., 1 = not relevant, 4 = highly relevant). Items with Item-Level Content Validity Index (I-CVI) below 0.8 (i.e., agreement by at least 4 out of 5 experts) were thoroughly reviewed. Based on the I-CVI scores and qualitative feedback from the experts, questions were either retained, reworded, or removed.

The initial tool consisted of 34 items related to AI perceived importance, AI perceived benefit, AI perceived risk, perceived ability, future directions, attitude, and AI readiness. However, the final tool was composed of 20 questions corresponding to four domains, as will be discussed later in this section. The responses to the questionnaire items were designed as a five-point Likert scale ranging from strongly disagree to strongly agree. Higher scores indicate greater agreement with the statement. Negatively worded items were reverse-coded prior to analysis so that higher domain scores consistently reflect higher levels of the underlying construct.

### Setting and Sampling

The target study population was consumers of the Jordanian mental healthcare system. The inclusion criteria included consumers with a mental health diagnosis who are eighteen years or older and mentally oriented as per the clinical judgment of the healthcare providers in charge. Those with no informed mental health diagnosis, under 18 years, or identified as disoriented were excluded.

The participants were recruited using convenience sampling. This sample included outpatient mental health clinic consumers at an educational hospital in the northern part of Jordan and four non-governmental mental health clinics in the Northern, Middle, and Southern regions of Jordan. A total of 431 questionnaires were collected for this study. The sample size exceeded the minimal required sample size according to G*power calculation (N = 204) and was comparable to previous studies in the same field, indicating that the study was sufficiently powered and enhancing the reliability and robustness of the statistical inferences

### Data collection methods

The process of data collection took place between July and September 2024. First, approvals to collect data from the International Medical Corps (IMC) and the hospital clinics were obtained from the Human Resources (HR) department and the institutional ethical review boards. Ethical approval for the study was granted by the Institutional Review Board of the Deanship of Research at Jordan University of Science and Technology (IRB # 6/168/2024) and the Mental Health and Psychosocial Support technical unit of the participating institutions. The study adhered to the principles of the Declaration of Helsinki.

The inclusion and exclusion criteria were shared with the focal points of the mental health clinics, who in turn shared a coded list of the clinics’ consumers who agreed to participate in the study, with a code assigned for each client by their case manager in addition to their mental health diagnosis. As part of the process to assess the capacity to provide informed consent. Formal cognitive assessments were conducted by the health care providers using the Mini-Mental State Examination (MMSE).

Structured interviews were conducted with the participants in each location between 8:00 am and 3:00 PM using digital forms by two research assistants with backgrounds in social sciences who were recruited for the purpose of data collection. During the interview, the data collectors introduced themselves, explained the research idea, and assured confidentiality and anonymity. Participants’ eligibility was confirmed before the questionnaire-filling process. All participants confirmed their voluntary participation in the study through online informed consent.

## Results

### Pilot Study: Reliability and validity testing

A pilot study (N = 23) was conducted prior to data collection. This sample size was deemed appropriate for this pilot study as it falls in an accepted range of 20–25 for studies aiming at evaluating the efficacy of an instrument in a single group [19]. [20] specifies that a minimum of 24 responses is adequate for calculating Cronbach’s alpha in a pilot study. However, considering the practical constraints and the exploratory nature of this pilot, a sample size of 23 was considered acceptable and sufficient for preliminary analysis. The primary purpose of this pilot phase was to evaluate the clarity, feasibility, and preliminary internal consistency of the initial questionnaire, rather than to establish definitive psychometric properties.

Cronbach’s alpha was used to evaluate the questionnaire’s internal consistency, and scores varied from 0.5 to 0.95 (Table 1). Given the small sample size (N = 23), these reliability estimates should be interpreted with caution, as Cronbach’s alpha may be unstable in small samples. Therefore, the pilot findings were treated as exploratory and used primarily to guide item refinement rather than to draw definitive conclusions about reliability.

**Table 1:** Cronbach’s alpha scores for subscales.

Scale	Subscale	Number of Items	Item range	Cronbach’s alpha
AIP	Perceived importance	4	1-4	.92
	Perceived benefit	5	5-9	.94
	Attitude	6	10-15	.50
	Readiness	4	16-19	.95
	Ability	4	20-23	.66
	Future directions	6	24-29	.89
	Perceived risk	5	30-34	.93
AIP total		34	1-34	.93

Because some domains did not achieve the minimal cut-off point of good internal reliability, further modifications and iterations were deemed necessary to refine the AIP tool as illustrated next.

### Principal component analysis

To explore the underlying dimensionality, reduce redundancy, and improve reliability, a factor reduction using Principal Component Analysis (PCA) was employed on the initial 34-item version of the instrument (N = 431). Four domains were identified with eigenvalues that are greater than one, a threshold based on Kaiser’s criterion, which states that only factors with eigenvalues above one should be retained, as they represent a meaningful amount of variance, explaining a total variance of 72.8% (Table 2).

**Table 2:** Principal Component Analysis (AIP).

Total Variance Explained						
Component	Initial Eigenvalues			Rotation Sums of Squared Loadings		
	Total	% of Variance	Cumulative %	Total	% of Variance	Cumulative %
1	7.021	35.105	35.105	4.131	20.655	20.655
2	4.812	24.058	59.164	3.681	18.405	39.060
3	1.443	7.213	66.377	3.611	18.056	57.116
4	1.294	6.471	72.847	3.146	15.731	72.847
5	.671	3.354	76.201			
6	.579	2.896	79.097			
7	.518	2.591	81.688			
8	.465	2.323	84.011			
9	.413	2.066	86.077			
10	.376	1.881	87.958			
11	.347	1.736	89.694			
12	.328	1.639	91.332			
13	.290	1.451	92.783			
14	.258	1.291	94.074			
15	.238	1.188	95.263			
16	.228	1.140	96.403			
17	.204	1.022	97.425			
18	.183	.914	98.338			
19	.173	.865	99.203			
20	.159	.797	100.000			

Extraction Method: Principal Component Analysis.

PCA is an exploratory technique and was used in this study to identify the latent structure of the instrument and guide item reduction. It was not intended as a confirmatory analysis. Future studies are recommended to validate this structure using Confirmatory Factor Analysis (CFA).

To improve interpretability, the Varimax rotation method was applied, a commonly used rotation that maximizes the loading of each variable on a single factor, thus simplifying the factor structure.

**Fig 2:**
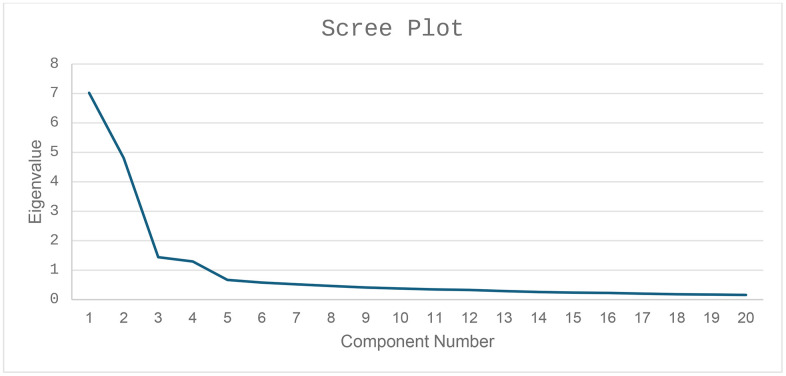
Scree plot for AIP.

Additionally, the number of retained components was further validated using a scree plot, which showed a clear inflection point (“elbow”) after the fourth component, supporting the 4-component structure (Fig 2).

Items were retained if they had factor loadings ≥ 0.40 on one component and did not cross-load significantly on more than one factor. Items not meeting this criterion were reviewed and either revised or removed to maintain conceptual clarity and construct distinctiveness.

Accordingly, the AIP items were regrouped and reduced. The final questionnaire consisted of 20 items divided evenly over 4 domains: AI Acceptance and Readiness (AIAR), AI Perceived Importance (AIPI), AI Perceived Risk (AIPR), and AI Perceived Challenges (AIPC).

After modifying the questionnaire, internal reliability tests were run. The results of the modified questionnaire’s internal reliability scores demonstrated very good to high reliability with Cronbach’s alpha values ranging between 0.85 and 0.92 (Table 3). Therefore, the modified version was used in the current study.

**Table 3:** Cronbach’s Alpha of the modified AIP.

No.	Variable	Cronbach’s Alpha	Number of Items
1	AI Acceptance and Readiness (AIAR)	0.92	5
2	AI Perceived Importance (AIPI)	0.92	5
3	AI Perceived Risk (AIPR)	0.90	5
4	AI Perceived Challenges (AIPC)	0.85	5

### Descriptive analysis of the demographic profile

The demographic profile of participants is summarized in Table 4. The sample (431 participants) consists of 55.7% females (n=240) and 44.3% males (n=191). Among the participants, 67.1% (n=289) are married, 23% (n=99) are single, 6.3% (n=27) are divorced, and 3.7% (n=16) are widowed. Most of the participants completed at least secondary school, and only about 32% (n= 134) had a diploma, bachelor’s, or postgraduate degree. The vast majority of the respondents (75.2%, n=324) reported a monthly income of less than 300 JD (equivalent to 423 USD). Around half of the respondents were born in Syria (52.4%, n=226), while 39.9% (n=172) were born in Jordan and only 7.7 % (n=33) born in other countries.

**Table 4:** Demographic Profile for Respondents in The Selected Mental Health Clinics (N=431).

Variable	Categories	Frequency	Percentage
Age	18-39	237	55.0
	40-59	176	40.8
	60+	18	4.2
Gender	Male	191	44.3
	Female	240	55.7
Marital Status	Single	99	23.0
	Married	289	67.1
	Divorced	27	6.3
	Widowed	16	3.7
Educational Level	Primary School	209	48.5
	Secondary School	88	20.4
	Diploma	22	5.1
	Bachelor’s Degree	87	20.2
	Postgraduate Degree	25	5.8
Monthly Income	<300 JOD	324	75.2
	300 JOD – 600 JOD	48	11.1
	600 JOD – 900 JOD	49	11.4
	>900 JOD	10	2.3
Place of Birth	Jordan	172	39.9
	Syria	226	52.4
	Other	33	7.7
Occupation	Student	35	8.1
	Unemployed	173	40.1
	Part-time job	48	11.1
	Full-time job	80	18.6
	Retired	8	1.9
	Permanently unable to work/ ill	87	20.2
Health Insurance	Yes	77	17.9
	No	354	82.1
Mental Health Diagnosis	Mood Disorders (Depression, BAD)	166	38.5
	Anxiety Disorders	125	29.0
	Psychotic Disorders (Schizophrenia)	14	3.2
	Obsessive-Compulsive Disorder (OCD)	38	8.8
	Trauma-Related Disorders (e.g., PTSD)	20	4.6
	Eating disorder	7	1.6
	Neurodevelopmental Disorders (e.g., Adult-ADHD)	18	4.2
	Others (e.g., somatic disorders, substance abuse, personality disorders.)	43	10.0

Participants were mostly unemployed (40.1 %, n=173) or permanently unable to work (20.2%, n=87) with only around 30% having full or part-time jobs. Only 17.9% (n=77) had health insurance. Most of the participants (64%, n=276) had at least one disability or chronic illness. The most common mental health conditions reported were mood disorders, such as depression and bipolar disorder (38.5%, n=166), anxiety disorders such as generalized anxiety disorder and panic disorder (29%, n=125), obsessive-compulsive disorder (8.8%, n=38), and others.

### Group differences and correlational analysis

This study showed no significant differences between participants’ demographic characteristics and their AIAR. For example, an independent samples t-test was conducted to examine differences in AIAR between male and female participants. The results showed no statistically significant difference between gender groups (F = 0.871, p = 0.351), indicating that AIAR did not differ by gender in this sample.

In addition, a Pearson correlation was conducted to assess the relationship between age and AIAR. The results indicated a non-significant positive correlation (r = 0.058, p = 0.228), suggesting that age was not significantly associated with AIAR.

### Descriptive analysis of the main study variables

Table 5 in this section shows the mean and standard deviation for each dimension of the AIP. With a mean score of 2.78 and a standard deviation of 0.87, the variable “perceived challenges” had the highest mean score. With a mean score of 2.18 and a standard deviation of 0.83, the variable “perceived importance and benefit” had the lowest mean score.

**Table 5:** Descriptive Analysis (Mean and Standard Deviation) of Study Variables.

No.	Variable	Min.	Max.	Mean	Std. Deviation
1	AIAR	1	5	2.7	0.96
2	AIPI	1	5	2.18	0.83
3	AIPR	1	5	2.58	0.92
4	AIPC	1	5	2.78	0.87

### Descriptive analysis of the items

Each of the items in acceptance and readiness, perceived importance and benefit, perceived risk, and perceived challenges is discussed separately in this section.

#### Descriptive analysis of readiness and acceptance.

A descriptive analysis of readiness and acceptance items is presented in Table 6. Item number 4, “Future health diagnoses will be made by an AI doctor,” had the highest mean score, with a mean value of 2.92 and a standard deviation of 1.16. Meanwhile, item number 5, “I see potential for technology applications and AI to enhance patient monitoring and follow-up,” had the lowest mean score, with a value of 2.53 and a standard deviation of 1.04.

**Table 6:** Descriptive Analysis (Mean and Standard Deviation) of AIAR.

No.	Item	Mean	Std. Deviation
1	Future health education and counseling will rely on technology applications and AI	2.72	1.11
2	Future group and individual therapy/support groups will be run by an AI	2.81	1.13
3	I expect that technology applications and AI will lead to better overall health outcomes for patients.	2.55	1.03
4	Future health diagnoses will be made by an AI doctor	2.92	1.16
5	I see potential for technology applications and AI to enhance patient monitoring and follow-up	2.53	1.04

#### Descriptive Analysis of Perceived Importance Items.

A descriptive analysis of Perceived Importance items is presented in Table 7. Item number 5, “ I think technology applications and AI can be great problem-solving tools,” had the highest mean score, with a value of 2.31 and a standard deviation of 1.0. Meanwhile, item number 4, “ I think technology applications and AI are important tools,” had the lowest mean score, with a value of 2.06 and a standard deviation of 0.9.

**Table 7:** Descriptive Analysis (Mean and Standard Deviation) of Perceived Importance Items.

No.	Item	Mean	Std. Deviation
1	I think technology applications AI can help me search for health-related information	2.16	.92
2	I think technology applications and AI are useful tools in healthcare	2.19	.96
3	I believe technology applications and AI can reduce waiting times and improve healthcare service efficiency	2.21	.98
4	I think technology applications and AI are important tools	2.06	.90
5	I think technology applications and AI can be great problem-solving tools	2.31	1.0

### Descriptive analysis of perceived risk items

A descriptive analysis of Perceived Risk items is presented in Table 8. Two items, number 3, “ I am concerned about the security of my health data when using technology applications and AI tools,” and 4 “I feel the healthcare system that uses AI is less caring and humane about me” had the highest mean score, with a value of 2.61 and a standard deviation of 1.1. Item number 5, “ I am afraid that technology applications and AI systems will someday replace health professionals,” had the lowest mean score of 2.52 and a standard deviation of 1.1.

**Table 8:** Descriptive Analysis (Mean and Standard Deviation) of Perceived Risk Items.

No.	Item	Mean	Std. Deviation
1	I am skeptical about relying on technology applications and AI for critical medical decisions.	2.56	1.1
2	I have reservations about sharing sensitive health data with technology applications and AI systems.	2.59	1.0
3	I am concerned about the security of my health data when using technology applications and AI tools.	2.61	1.1
4	I feel the healthcare system that uses AI is less caring and humane about me	2.61	1.1
5	I am afraid that technology applications and AI systems will someday replace health professionals	2.52	1.1

#### Descriptive analysis of perceived challenges-AI items.

A descriptive analysis of the perceived challenges items is presented in Table 9. Item number 1, “People who like technology applications and AI are reserved and antisocial,” had the highest score with a mean value of 2.91 and a standard deviation of 1.1 while item number 5, “I resent the thought of having to deal with technology applications and AI instead of healthcare professionals,” had the lowest score with a mean value of 2.71 and a standard deviation of 1.1

**Table 9:** Descriptive Analysis (Mean and Standard Deviation) of Perceived Challenges.

No.	Item	Mean	Std. Deviation
1	People who like technology applications and AI are reserved and antisocial.	2.91	1.1
2	I feel nervous when I think of using technology applications and AI	2.72	1.1
3	Health-related online groups, forums, and discussions are a waste of time	2.83	1.0
4	Technology applications and AI are too complicated for me to learn well	2.79	1.2
5	I resent the thought of having to deal with technology applications and AI instead of healthcare professionals	2.71	1.1

## Discussion

This study aimed to examine the psychometric properties of the AIP as well as provide baseline assessment data for the perception of AI from the perspectives of the main beneficiaries of its application (i.e., mental health consumers). This is the first study to assess the AI’s perception among mental health consumers in Jordan. The findings of the psychometric analyses demonstrated excellent internal consistency, evidenced by high Cronbach’s alpha scores across all domains that exceed the threshold of good-excellent reliability [21]. This strong reliability indicates that the AIP tool consistently measures AI’s perception. The structural validity of the tool was supported by the panel of experts’ evaluation and the PCA, which showed that each domain captured distinct and relevant aspects of AI’s perception. The PCA also revealed that 72.8% of the variance was explained by these factors, exceeding the commonly accepted threshold for adequate variance explained by the principal components [22].

The description of the demographic data presented in the results section highlights the complex psycho-socioeconomic context of this population, summarized in poverty, lack of employment, and limited education. Social determinants of mental health among refugees were reported to be common by a large body of literature [23]. Previous studies shed light on the role of the importance of addressing income and work-related problems among Syrian refugees in Jordan [24]. This study showed no significant differences between participants’ demographic characteristics and their AIAR, which is consistent with the findings of [25], who showed that age, gender, and educational level did not predict attitudes toward AI. Similarly, [26] found that age did not have any impact on the perception of AI’s future. Additionally, some research revealed a relationship between AI readiness and acceptance and gender but not age or job status [27]. According to [28], even though the gender gap is still present when considering attitudes toward technology use, this gap has shown a small reduction over time, and this reduction was particularly noticeable in women’s

In this study, the mean score for acceptance and readiness (AIAR) suggests a moderate level, indicating some level of consumers’ openness to interact with AI in their health care, but not a particularly strong or enthusiastic acceptance and readiness. These results are compatible with other research assessing the readiness of patients/consumers in other health sectors [25,29]. For example, an assessment of the readiness of primary healthcare consumers during the COVID-19 pandemic reported average readiness toward adopting AI technologies in healthcare [25].

The term “moderate” was used based on the interpretation of a 5-point Likert scale where 3 is the neutral midpoint. Scores ranging from 2.5 to 3.5 were considered moderate, while scores less than 2.5 were considered low perception, and those above 3.5 were considered high perception.

The findings of this study revealed that the participants perceived AI in mental health as moderately important, indicating a lack of comprehensive recognition of its significance. Additionally, the participants showed a moderate level of AI perceived risk (AIPR), which signals the presence of some concerns regarding the potential negative impact of AI in healthcare. The level of AI perceived challenges (AIPC) was also moderate, which reveals that participants perceive some barriers concerning dealing with AI in mental health, such as perceptions of technological complexities and fear of social isolation. Therefore, future implementation of AI in the mental health sector should be strategically planned, taking into consideration the possible limitations and challenges to effectively enhance utilization.

The findings of this study offer several practical and policy-level implications. For mental health providers, assessing the level of AI acceptance, perceived importance, perceived challenges, and risks can inform the implementation and design of awareness and training programs to bridge identified gaps in any of the four constructs. For example, mental health professionals could incorporate brief AI-focused education into their sessions, highlighting the supportive and beneficial aspects of AI in mental health care, which may enhance their familiarity, increase their perception of its importance, and mitigate their perception of risks and challenges.

For policymakers, the insights gained from the deployment of this tool can emphasize the need for drafting comprehensive digital health policies that address the concerns and capabilities of vulnerable populations like consumers with mental health conditions.

Additionally, the findings of this study can also offer insight for technology development companies by emphasizing the need to customize certain characteristics in the AI tools to address the specific needs of certain groups. For example, considering a more user-friendly interface for populations with higher levels of perceived challenges, and more assurance of privacy for populations with higher levels of perceived risks.

### Strengths and limitations

This is the first study to assess the level of AI perception among mental health consumers in Jordan. The respondents were recruited from different locations and mental health sectors in Jordan; however, further analysis of the AIP psychometrics in other communities/populations and across different health sectors is needed. Additionally, the study deployed a convenience sampling technique, which may influence the results’ generalizability, and a cross-sectional design, which may affect the causal inference. Moreover, although PCA was conducted on an adequate sample, CFA was not performed; therefore, the factor structure should be further validated in future studies.

## Conclusion

This study provides evidence that the AIP tool is both valid and reliable for the assessment of AI’s perception among mental health consumers in Jordan. The findings of this study indicate that the AIP tool can effectively assess the AIAR, which highlights its potential in informing strategies for AI adoption in the mental health sector. The results of the current study revealed that mental health consumers in Jordan have a moderate level of AI perception, which provides evidence to guide health organizations’ leaders, supervisors, and policymakers in their planned actions and policies to integrate AI technologies into the mental health sector.

## Supporting information

S1 File Row data. Raw anonymized dataset used for the analyses reported in this study.
(XLSX)

S2 File The tool. The study questionnaire used for data collection, including all questionnaire items and response options.
(DOCX)
