# Supplementary material for: Development of a scale for measuring the perception of artificial intelligence among mental health consumers
Source: PLoS One. 2026 Jul 30;21(7):e0354305. doi: 10.1371/journal.pone.0354305 (PMC13422870; doi:10.1371/journal.pone.0354305)
Supplement: S2 File — The study questionnaire used for data collection, including all questionnaire items and response options. (DOCX) [file pone.0354305.s002.docx]

**Artificial Intelligence Perception (AIP) Questionnaire**

As technology and AI continue to advance, they are becoming an integral part of our daily lives. We invite you to participate in this survey that aims to understand your perception of the adoption of AI tools in healthcare.

The survey takes approximately **5 minutes** to complete, and all responses will be **kept completely confidential.**

Participation is **voluntary,** and your completion of the survey indicates your consent. Your contribution will help benefit both the Jordanian community and the broader field of scientific research.

Choose the response that best reflects your belief or attitude for each statement

|  | | Disagree  Strongly | Disagree | Not certain | Agree | Agree Strongly |
| --- | --- | --- | --- | --- | --- | --- |
| **F1: AI Readiness and Acceptance** | | | | | | |
|  | Future health diagnoses will be made by an AI doctor |  |  |  |  |  |
|  | Future group and individual therapy/support groups will be run by an AI |  |  |  |  |  |
|  | Future health education and counseling will rely on technology applications and AI |  |  |  |  |  |
|  | I see potential for technology applications and AI to enhance patient monitoring and follow-up |  |  |  |  |  |
|  | I expect that technology applications and AI will lead to better overall health outcomes for patients. |  |  |  |  |  |
| **F2: AI Perceived Importance and Benefits** | | | | | | |
|  | I think technology applications and AI are important tools |  |  |  |  |  |
|  | I think technology applications and AI are useful tools in healthcare |  |  |  |  |  |
|  | I think technology applications and AI can be great problem-solving tools |  |  |  |  |  |
|  | I think technology applications and AI can help me search for health-related information |  |  |  |  |  |
|  | I believe technology applications and AI can reduce waiting times and improve healthcare service efficiency. |  |  |  |  |  |
| **F3. AI Perceived Risk** | | | | | | |
|  | I am concerned about the security of my health data when using technology applications and AI tools. |  |  |  |  |  |
|  | I have reservations about sharing sensitive health data with technology applications and AI systems. |  |  |  |  |  |
|  | I am skeptical about relying on technology applications and AI for critical medical decisions. |  |  |  |  |  |
|  | I feel the healthcare system that uses AI is less caring and humane toward me. |  |  |  |  |  |
|  | I am afraid that technology applications and AI systems will someday replace health professionals. |  |  |  |  |  |
| **F4. AI Perceived Challenges** | | | | | | |
|  | I feel nervous when I think of using technology applications and AI. |  |  |  |  |  |
|  | People who like technology applications and AI are reserved and antisocial. |  |  |  |  |  |
|  | I resent the thought of having to deal with technology applications and AI instead of healthcare professionals. |  |  |  |  |  |
|  | Health-related online groups, forums, and discussions are a waste of time. |  |  |  |  |  |
|  | Technology applications and AI are too complicated for me to learn well. |  |  |  |  |  |
